# Evaluation and perspectives of the OrphanAnesthesia project – a survey among anesthesiologists in Germany

**DOI:** 10.1186/s12871-026-03665-7

**Published:** 2026-02-11

**Authors:** Christine Gaik, Tino Münster, Franz-Josef Kretz, Philipp Gude

**Affiliations:** 1 Philipps University of Marburg, Marburg, Germany; 2 Department of Anesthesiology and Intensive Care Medicine, University Hospital Giessen and Marburg, Campus Marburg, Marburg, Germany; 3 Department of Anesthesiology and Intensive Care Medicine, Krankenhaus Barmherzige Brüder, Regensburg, Germany; 4 Department of Anesthesiology and Intensive Care Medicine, Olgahospital (Former head), Stuttgart, Germany; 5 Department of Anesthesiology and Intensive Care Medicine, St. Josef and St. Elisabeth Hospital, Ruhr University Bochum, Bochum, Germany

**Keywords:** Rare diseases, Perioperative care, Anesthesia, Anesthesiology, Patient safety

## Abstract

**Background:**

Patients with rare diseases face unique risks during anesthesia due to the multisystemic nature of their conditions and the limited availability of evidence-based guidance. In most cases, perioperative guidelines tailored to either the underlying disease or anesthetic management are lacking, leaving anesthetists with considerable uncertainty in clinical decision-making. The OrphanAnesthesia project, initiated in 2005 by the Scientific Working Group on Pediatric Anesthesia of the German Society for Anesthesiology and Intensive Care Medicine, addresses this gap by providing structured, peer-reviewed anesthesia recommendations for rare diseases. This study aims to assess current status, clinical relevance, and future perspectives of the OrphanAnesthesia platform among anesthetists in Germany.

**Methods:**

A cross-sectional online survey was conducted among members of the German Society for Anesthesiology and Intensive Care Medicine and the German Association of Anesthetists from February to March 2025. The anonymous questionnaire included 17 items covering awareness, accessibility, frequency of use, evaluation of content, and future needs related to the OrphanAnesthesia platform. Descriptive statistics were used to analyze the data.

**Results:**

Out of 2,098 responses, 1,921 fully completed surveys were included in the final analysis (response rate: 8.1%). A total of 81% of respondents were familiar with OrphanAnesthesia; of these, over half accessed the platform five or more times annually. The recommendations were rated as useful by 93% of users, and 75% considered them to be up to date. 22% of all respondents were already familiar with the emergency cards introduced in 2022, and 83% of those rated them as ‘good’ or ‘very good’. 90% of respondents supported the development of a dedicated anesthesia problem card for patients with rare diseases. 72% indicated that a mobile app would increase their use of the platform, and 91% believed it could improve patient safety.

**Conclusion:**

The OrphanAnesthesia project is well known among German anesthetists and is considered a valuable resource in the perioperative care of patients with rare diseases. To further enhance its impact, targeted improvements should include greater awareness of existing tools (e.g., emergency cards), the development of individualized anesthesia problem cards, and mobile solutions. These efforts may support evidence-based, safer and more individualized anesthesia care in patients with rare diseases.

**Supplementary Information:**

The online version contains supplementary material available at 10.1186/s12871-026-03665-7.

## Background

A disease is considered rare within the European Union (EU) if it affects no more than 5 individuals per 10,000 of the general population [1]. In contrast, the United States Food and Drug Administration (FDA) defines a rare disease as one affecting fewer than 200,000 individuals nationwide [2]. Although individually uncommon, rare diseases collectively impact over 300 million people worldwide, corresponding to a population prevalence of up to 5.9%, with approximately 70% of cases manifesting in childhood [3–5]. This represents a major challenge to healthcare systems as well as in perioperative care.

Anesthetists working across a broad range of clinical environments – including academic medical centers, regional hospitals, outpatient facilities, and emergency medical services (EMS) – are increasingly likely to care for patients living with a rare disease. These patients are frequently at increased risk of perioperative and anesthesia-associated complications, not only due to the complexity and multisystem nature of their underlying conditions, but also owing to the lack of disease-specific, anesthesia-related evidence to guide clinical decision-making [6, 7].

To address this critical knowledge gap, the Scientific Working Group on Pediatric Anesthesia of the German Society for Anesthesiology and Intensive Care Medicine (DGAI) initiated the OrphanAnesthesia project in 2005. The project provides structured, scientifically based, peer-reviewed recommendations for the perioperative care of individuals with rare diseases. Each recommendation undergoes a dual peer-review process – by a disease expert and a specialist in anesthesiology – ensuring both scientific validity and clinical applicability.

The present study aims to assess the current status and perceived value of the OrphanAnesthesia project among anesthetists in Germany. Specifically, it investigates the level of awareness, accessibility, frequency of use, and perceived quality and usability of the online platform. Furthermore, it explores future expectations regarding the platform’s role in supporting clinical anesthesia practice for patients with rare diseases.

## Methods

### Ethics approval and setting

This cross-sectional study was conducted among members of the DGAI and the German Association of Anesthetists (BDA) from February 2025 to March 2025. Ethics approval for the study was prospectively granted by the Ethics Committee of the Medical Faculty of the Philipps University of Marburg (reference number 25–42 ANZ, granted on 10 February 2025). This manuscript adheres to the current Strengthening the Reporting of Observational Studies in Epidemiology (STROBE) guidelines.

### Participants

Members of DGAI and BDA were invited to take part in an anonymous online survey. Participation was entirely voluntary, with no financial or other incentives offered. The survey invitation was emailed to DGAI and BDA members on 28 February 2025 (Rare Disease Day), followed by a reminder on 17 March 2025.

### Study design

This survey was developed using a secure web-based platform (SurveyMonkey; San Mateo, California, USA) and comprised 17 questions, several of which employed conditional logic. In these cases, follow-up questions were displayed only if specific responses met predefined criteria. Participants were invited to complete the survey via a link that directed them to the study homepage on the SurveyMonkey platform. The survey commenced with a brief introduction detailing the study’s aims and objectives, followed by an informed consent statement. Participation required respondents to actively confirm consent by selecting the appropriate option. To ensure data integrity, web cookies were employed to limit responses to one per respondent. All participants received the questions in an identical sequence. The complete questionnaire is provided in the supplementary material.

### Data management and analysis

Data management and analysis were performed using Excel 2013 (Microsoft Corporation, Redmond, WA, USA). Only fully completed questionnaires were included in the final analysis. Progression through the survey required responses to all preceding questions. However, certain subsequent questions were only shown if the previous response met predefined criteria. Consequently, the number of respondents varies by question and is reported accordingly. Descriptive statistics, including absolute and relative frequencies, were used to summarize the responses. The data represent a convenience sample, and no formal sample size calculation was conducted.

## Results

The initial survey invitation was emailed to 23,606 DGAI and BDA members, of whom 9,694 opened the email (41% open rate). A reminder was sent to 23,573 DGAI and BDA members, with 8,459 opening the email (36% open rate). In total, we received 2,098 responses. Of these, 177 questionnaires were incomplete and therefore excluded from the analysis. Consequently, 1,921 fully completed questionnaires were included in the final analysis, resulting in a response rate of 8.1%.

### Respondent characteristics and clinical focus

Respondents’ demographics, job positions, current workplaces, and work experience are summarized in Table 1.

**Table 1:** Data on respondents’ demographics

	All respondents (*n* = 1,921)	
	*n*	%
Job position		
Resident	216	11
Consultant	631	33
Senior consultant	718	37
Chief position	167	9
Other position	189	10
Workplace / Hospital of the respondents		
Hospital with less than 299 beds	296	15
Hospital with 300 to 499 beds	367	19
Hospital with 500 to 799 beds	334	17
Hospital with more than 800 beds and / or university hospital	565	29
Anesthetist in private practice	208	11
Freelance anesthetist	62	3
Other workplace	89	5
Experience as anesthetist		
Less than 5 years	125	7
5 to 10 years	285	15
11 to 20 years	571	30
For more than 20 years	940	49

The surveyed respondents reported that, over the past three years, 71% (1,361/1,921) primarily cared for adult patients, 20% (382/1,921) cared for both adult and pediatric patients equally often, 5% (96/1,921) primarily cared for pediatric patients, and 4% (82/1,921) had not cared for any pediatric patients.

### Accessing information on rare disease anesthesia

Participants were asked to indicate their current preferred sources of information regarding the anesthesiologic care of patients with rare diseases. Each respondent could select up to two options. The majority of respondents (67%, 1,296/1,921) identified the internet platform ‘OrphanAnesthesia’ as their primary source of information. ‘Internet search engines (e.g., Google, Bing)’ were used by 40% (766/1,921), while 33% (631/1,921) reported seeking ‘advice from colleagues or experts’. ‘Scientific databases such as PubMed or Orphanet’ were cited by 31% (600/1,921). ‘Textbooks’ were preferred by 14% (273/1,921), and 2% (41/1,921) selected the option ‘Other’.

### Awareness and utilization of the OrphanAnesthesia project

A total of 81% (1,561/1,921) of respondents reported being aware of the OrphanAnesthesia project, whereas 19% (360/1,921) indicated that they were not familiar with it. Respondents who were familiar with the OrphanAnesthesia project were then asked how frequently they had accessed the platform within the past three years. A total of 23% (359/1,562) reported visiting the website ‘more than ten times per year’, 33% (517/1,562) accessed it ‘five to ten times per year’, and 39% (611/1,562) ‘one to four times per year’. 5% (75/1,562) stated that they had ‘never’ visited the OrphanAnesthesia website at all during the last three years. Participants were also asked whether they were able to access the OrphanAnesthesia online platform at their current workplace if needed. In total, 66% (1,273/1,921) indicated that they could access the website via a ‘hospital computer or tablet’. Another 30% (585/1,921) reported that access was ‘only possible using their personal mobile device’, while 3% (63/1,921) stated that they were ‘unable to access the platform at their workplace at all’.

### Evaluation of the OrphanAnesthesia recommendations

To gain insights into the perceived quality and usefulness of the recommendations, participants who had previously accessed information from the OrphanAnesthesia online platform at least once were subsequently asked to evaluate the recommendations for the anesthesiologic care of patients with rare diseases. They were presented with a series of statements and asked to rate their level of agreement using a five-point scale: ‘strongly agree’, ‘agree’, ‘hardly agree’, ‘disagree’, or ‘I don’t know’.

**Fig. 1:**
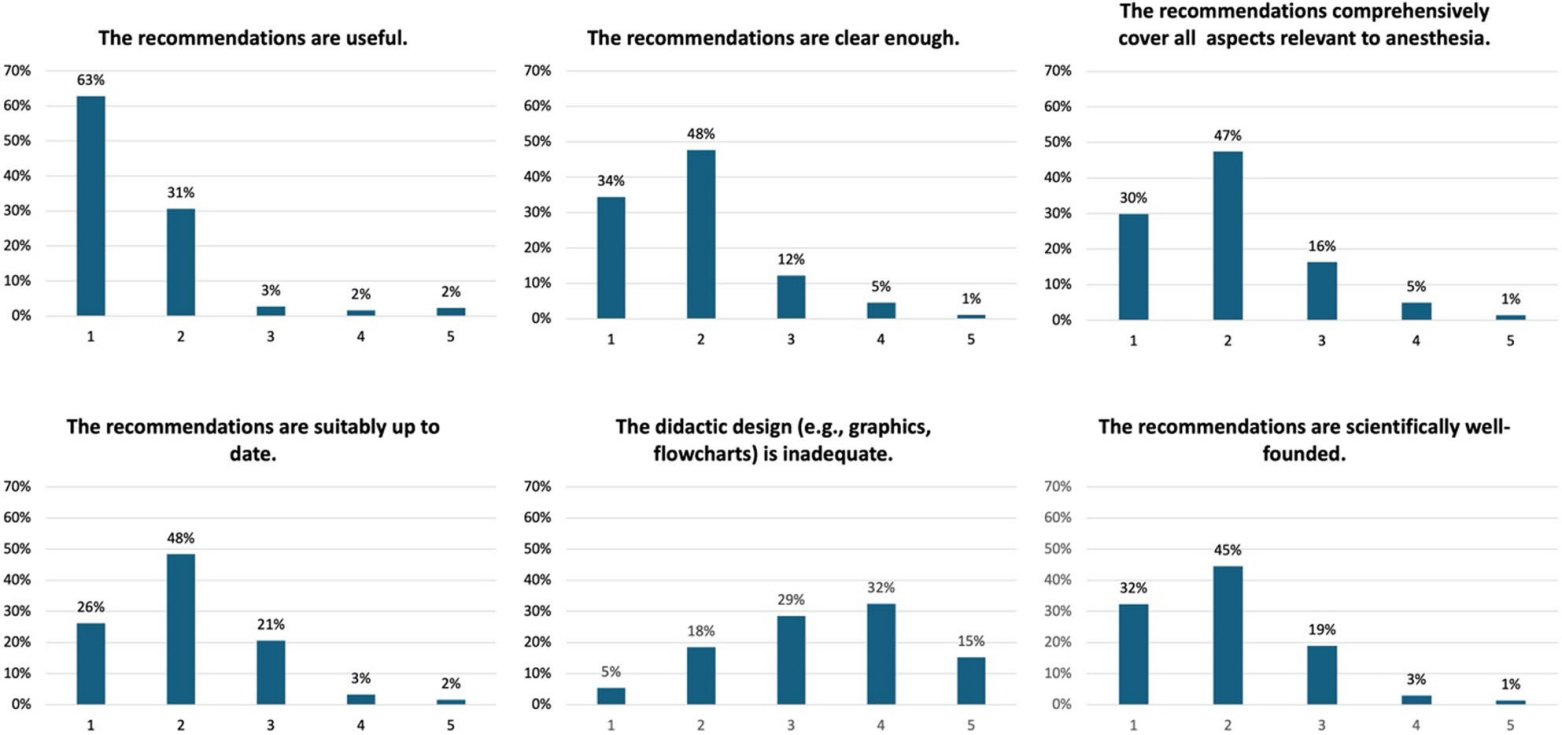
Detailed data on respondents’ evaluation of the quality and usefulness of the OrphanAnesthesia recommendations, assessed using a five-point Likert scale: 1 = ‘strongly agree’, 2 = ‘agree’, 3 = ‘hardly agree’, 4 = ‘disagree’, 5 = ‘I don’t know’ (*n* = 1,487 for each question)

93% (1,389/1,487) ‘strongly’ agreed with the statement that ‘the recommendations are useful’. 2% (24/1,487) either ‘hardly agreed’ or ‘did not agree’, and 3% (40/1,487) selected ‘I don’t know’. The statement that the recommendations are up to date was ‘(strongly) agreed’ with by 75% of respondents (1,108/1,487). In contrast, 3% (49/1,487) ‘hardly agreed’, 2% (24/1,487) ‘did not agree’, and 21% (306/1,487) selected ‘I don’t know’. Figure 1 displays the participants’ detailed responses to further evaluation statements on the recommendations.

### Knowledge of emergency cards provided in the recommendations

Respondents were asked whether they were familiar with the disease-specific emergency cards included in the recommendations for action. A total of 22% (431/1,921) answered ‘yes’, while 78% (1,492/1,921) indicated that they were not familiar with them. Those who were familiar with the emergency cards were subsequently asked to evaluate their usefulness. Of these, 29% (127/431) rated the cards as ‘very good’, 54% (231/431) as ‘good’, 13% (54/431) as ‘satisfactory’, and 4% (18/431) as ‘sufficient’. 0.2% (1/431) rated the cards as ‘poor’, and none selected the option ‘unsatisfactory’.

### Perspectives on a specialized anesthesia passport for rare diseases

Furthermore, respondents were asked to evaluate the perceived usefulness of a dedicated anesthesia passport for patients with a specific rare disease. The proposed tool was intended to provide concise, disease- and patient-specific information, including relevant medications, contraindications, anesthetic considerations, potential intraoperative risks, and individualized perioperative management strategies. A total of 55% (1,057/1,921) considered such a passport to be ‘very helpful’, 35% (666/1,921) ‘helpful’, 5% (69/1,921) ‘not very helpful’, and 1% (9/1,921) ‘not helpful at all’. An additional 6% (120/1,921) selected the option ‘I don’t know’.

### Evaluation of an OrphanAnesthesia newsletter

**Fig. 2:**
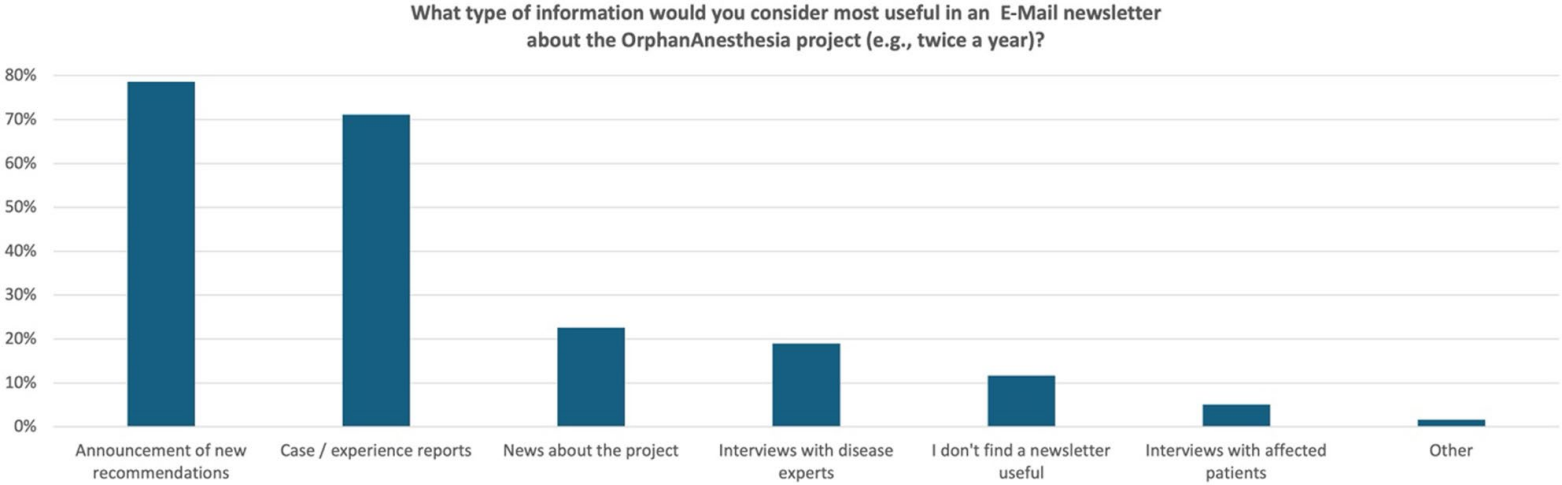
The figure presents potential topics for an OrphanAnesthesia newsletter that respondents rated as useful. A maximum of three options could be selected per respondent. (*n* = 1,921)

Survey participants were asked to indicate which types of content in a currently not existing E-Mail newsletter (e.g., published twice a year) about the OrphanAnesthesia project would be useful for them. They could select up to three predefined options and add a free-text response. Figure 2 illustrates the distribution of responses to this question.

### Functions and relevance of an app for the anesthetic management of rare diseases

**Fig. 3:**
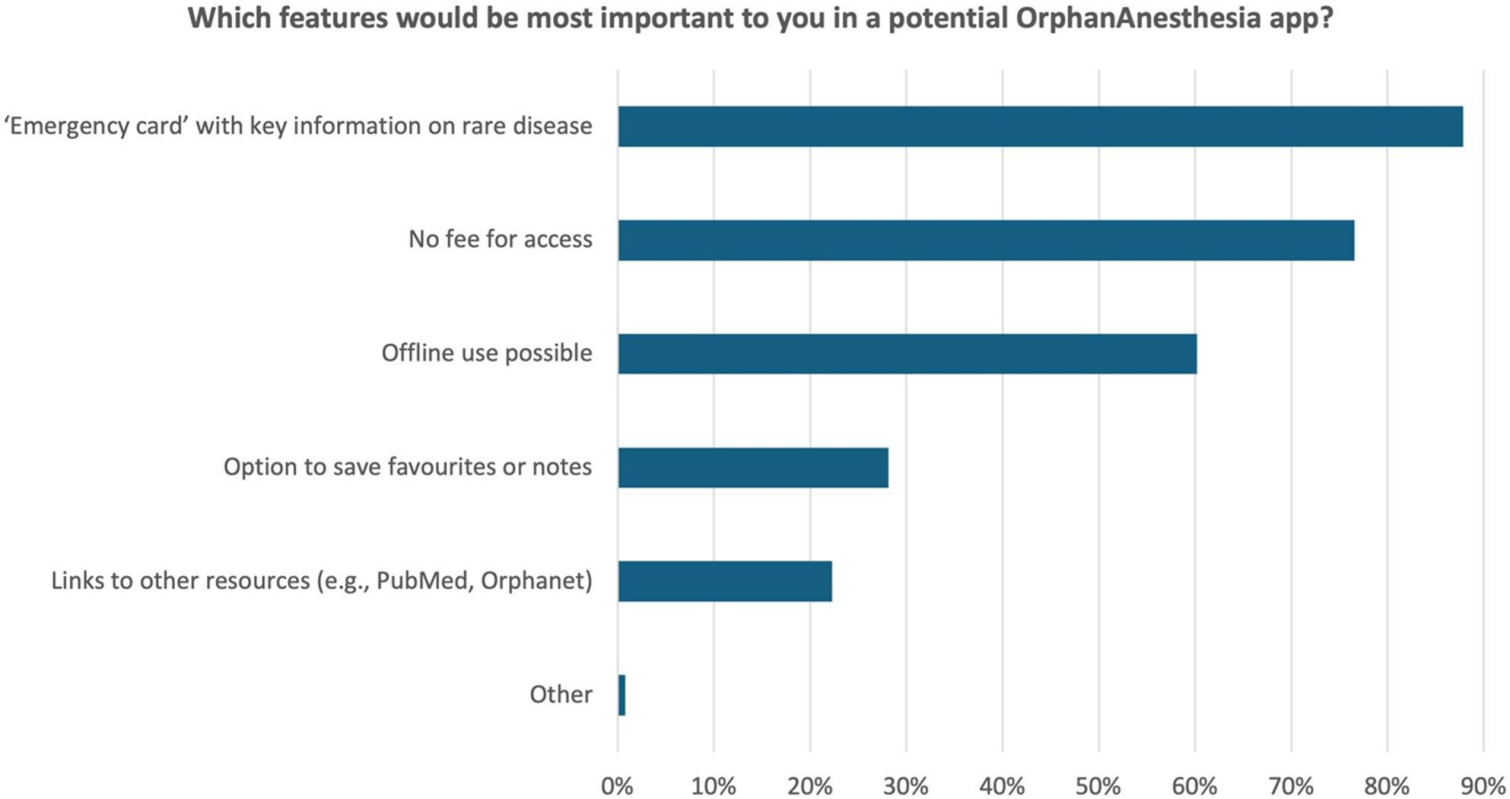
The figure illustrates potential features of an OrphanAnesthesia app that were rated as useful by respondents. A maximum of three options could be selected per participant (*n* = 1,391)

The final section focused on perceived benefits of a mobile app version of the OrphanAnesthesia website. First, respondents were asked whether the availability of an OrphanAnesthesia app would influence their usage behavior. A total of 72% (1,390/1,921) stated that they ‘would use OrphanAnesthesia more frequently if an app were available’, while 28% (531/1,921) indicated that ‘an app would not change their behavior’. Figure 3 illustrates the app functionalities deemed most important by respondents. 91% (1,269/1,391) of respondents believed that an OrphanAnesthesia app ‘could increase patient safety in the operating room’. 1% (11/1,391) ‘disagreed’ and 8% (111/1,391) selected the ‘I don’t know’ option.

## Discussion

This study provides a comprehensive national assessment regarding awareness, utilization, and perceived value of the OrphanAnesthesia project among anesthetists in Germany. The high number of fully completed responses (*n* = 1,921) underscores the relevance of this platform and offers valuable insights into its perceived utility in the context of rare disease anesthesia. The findings highlight the platform’s high visibility within the anesthesiology community and its relevance as a trusted, practice-oriented resource for perioperative care in patients with rare diseases.

The evolution and dissemination of the OrphanAnesthesia project provide important contextual background for interpreting the results of the present survey. OrphanAnesthesia was developed in collaboration with Orphanet (www.orpha.net), an internationally recognized online portal for rare diseases and orphan drugs. Since its foundation in 1997 at the Institut National de la Santé et de la Recherche Médicale (INSERM) in Paris, Orphanet has grown into a pan-European network supported by the European Commission, now active in more than 40 countries [8]. This established international infrastructure has supported the structured development and dissemination of OrphanAnesthesia recommendations over time. The milestones summarized in Table 2 illustrate the stepwise evolution of the project from its initiation in 2005 to its current scope and highlight its increasing integration into existing rare disease networks.

**Table 2:** Timeline of ‘milestones’ in the development of the OrphanAnesthesia project

2005	Initiation of the project and start of the database creation [9]
2011	Online presence and database of the project (www.orphananesthesia.eu) [6]
2014	Publication of two quotable recommendations per month as supplements to the journal ‘Anästhesiologie & Intensivmedizin’ (A&I) [9]
2016	Availability of a free browser-based app
2016	Download option for the patient safety card
2022	Addition of the emergency card as part of the recommendations [10]
2025	236 recommendations online (December 2025), some available in up to 6 languages

To date, OrphanAnesthesia has published over 230 disease-specific anesthesia recommendations and continues to expand its international network of experts and reviewers. Despite this progress, the primary goal of OrphanAnesthesia remains unchanged: to improve patient safety by providing peer-reviewed, evidence-based guidance for the perioperative management of patients with rare diseases. Given that more than 7,000 known rare diseases are currently known, the development of comprehensive anesthesia-specific guidance remains an ongoing and complex endeavor [3, 11].

**Fig. 4:**
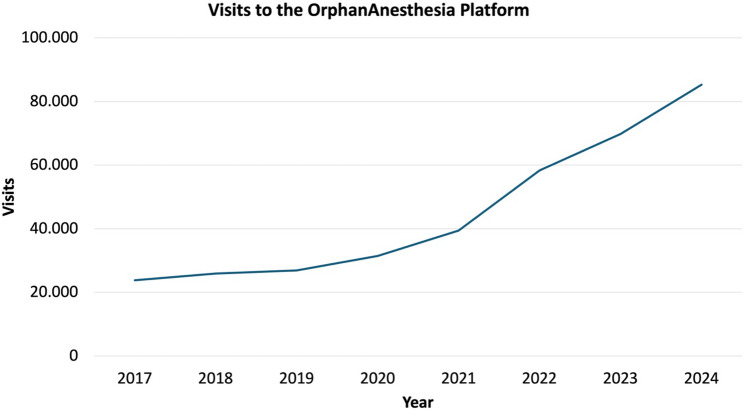
Overview of the number of visits of the online database OrphanAnesthesia in recent years since 2017. Data presented are based on internal usage statistics

**Fig. 5:**
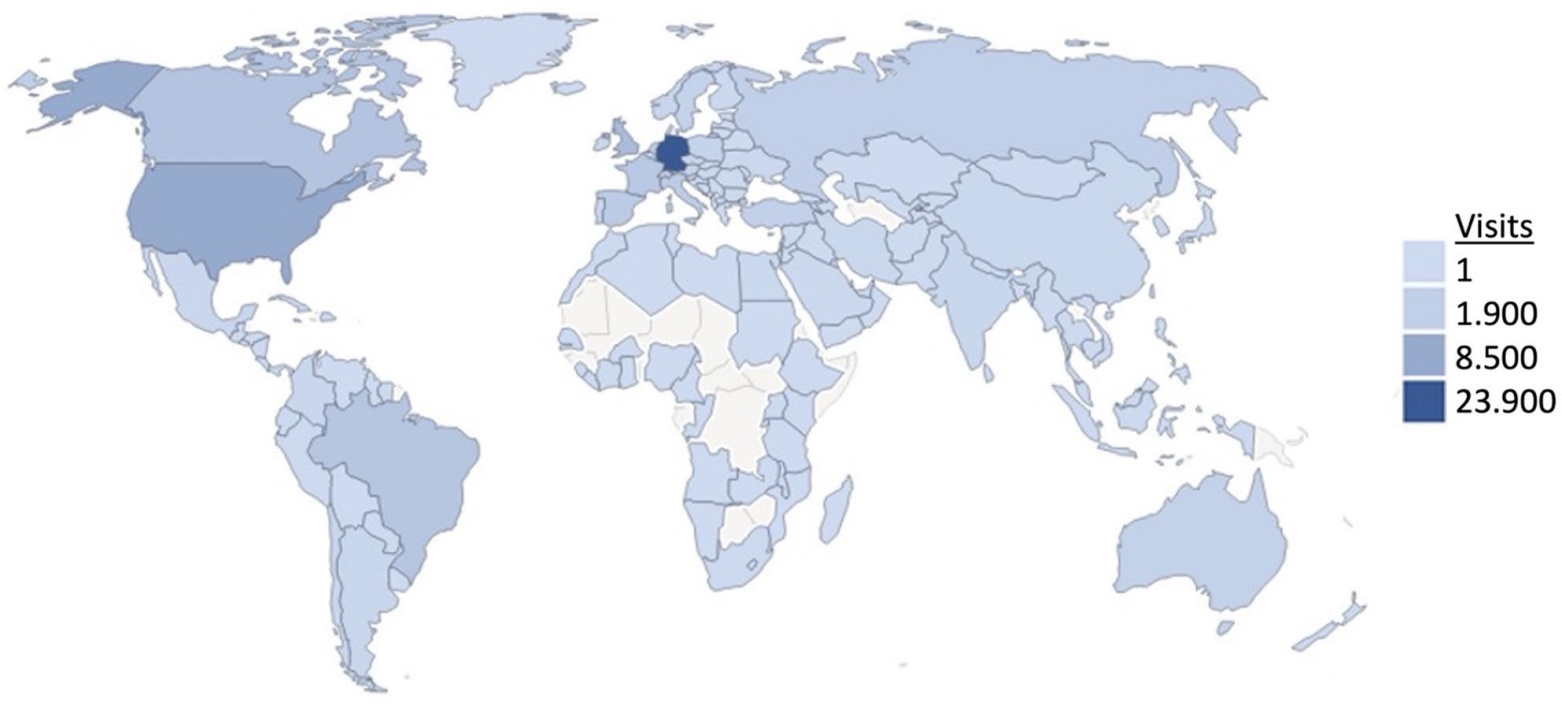
Global reach of the OrphanAnesthesia online platform. In 2024, the website was accessed by a total of 82,789 users from around the world, not limited to Germany. Darker shades of blue indicate a higher number of visits (see legend for details). Data presented are based on internal usage statistics

Usage data from the OrphanAnesthesia website further illustrate the growing relevance of the platform. As shown in Figs. 4 and 5, increasing website visits and a broad international user distribution underscore the increasing demand for reliable perioperative recommendations in the context of rare diseases (Figs. 4 and 5). In this context, the high level of awareness and frequent use reported by German anesthetists in the present survey appear plausible and support the interpretation of the study findings.

The survey encompassed a broad, yet predominantly experienced cohort: nearly half of respondents reported more than 20 years of professional experience, and most held consultant-level positions or higher. Participants represented a wide range of healthcare settings, with approximately half working in hospitals with 500 or more beds or in university hospitals It is plausible that participation was more common among experienced anesthetists and those affiliated with tertiary care institutions, where multidisciplinary teams and complex case profiles are more frequently encountered. Such settings are also more likely to manage patients with rare diseases, which may account for the comparatively high levels of awareness and use of resources such as OrphanAnesthesia.

Many syndromic entities and rare diseases are incompatible with life or significantly reduce life expectancy, resulting in a high proportion of affected individuals not surviving into adulthood. Consequently, patients with these conditions are more frequently managed by pediatric anesthetists rather than general anesthetists [12]. Findings from our study indicate that the majority of respondents primarily treat adult patients, while 20% regularly manage both adult and pediatric cases, and only 5% focused predominantly on pediatric patients. These proportions align with national data, which indicate that of the roughly 15 million anesthetic procedures performed annually in Germany, only around 400,000 (approximately 3%) involve children under the age of five [13, 14]. Given this distribution, it is less likely for anesthetists – especially those not working in specialized centers – to routinely manage pediatric anesthesia. In line with current recommendations, anesthetic care for children, particularly those with complex or rare conditions, should ideally be provided by anesthetists with specific expertise in pediatric anesthesia working with experienced teams in institutions with age-appropriate structures and processes [15, 16].

Anesthetists are responsible for the management of patients during a brief yet critical period, characterized by profound physiological changes and elevated risk for complications [17]. Regarding that perioperative care providers often have limited experience with specific rare diseases, access to reliable, high-quality, and disease-specific information is essential. In addition to patient history and information provided by caregivers, anesthetists ideally rely on prior diagnostic findings, discharge summaries, and detailed anesthesia records to inform perioperative planning. However, supplementary resources such as specialized textbooks are often not readily available at the point of care due to time constraints and limited accessibility [11]. While open internet searches can generate large volumes of information, these results frequently require critical evaluation with regard to quality, accuracy, and clinical applicability [11]. The ability to appraise and apply information from predominantly online sources is therefore increasingly important – particularly in light of the limited availability of robust, evidence-based data in the field of rare disease anesthesia [11, 18]. Although databases such as PubMed, Online Mendelian Inheritance in Man (OMIM), Genetic and Rare Disease Information Center (GARD) and Orphanet provide valuable disease-specific insights, they often lack content that is directly relevant in the context of anesthetic management [11, 12, 17]. OrphanAnesthesia appears to address this need by offering structured, peer-reviewed, and practice-oriented recommendations tailored to the requirements of anesthetists [6]. Consistent with this, our survey identified the platform as the most frequently used source of information for anesthesia in patients with rare diseases – surpassing search engines, peer consultation, and general scientific databases. This reflects the high level of trust in the platform’s clinical relevance and quality in the field of rare disease anesthesia.

With 81% of respondents reporting familiarity with OrphanAnesthesia, the platform demonstrates significant visibility within the anesthesiology community. Notably, actual usage varied: while over half of those familiar with the platform accessed it at least five times per year, others used it only occasionally, and 5% had not accessed it at all in the past three years despite being aware of it. The high rate of reported access – 66% via hospital devices and 30% via personal mobile devices – suggests substantial integration into clinical practice. However, the fact that nearly one-third of respondents reported no institutional access highlights a gap in digital infrastructure that could hinder timely information retrieval.

Given the pace and complexity of perioperative care, the availability of a mobile app – ideally with offline functionality – could significantly enhance the platform’s clinical utility. Survey results support this, with the majority of respondents indicating that a mobile app would increase their use of the platform. Importantly, 91% believed that such an app could improve patient safety in the operating room. These findings underscore the need for user-friendly, point-of-care tools that provide rapid access to reliable, disease-specific anesthesia guidance – similar to other digital initiatives led by national professional societies [19, 20].

Evaluation of the OrphanAnesthesia recommendations themselves revealed a high level of acceptance and perceived value. A total of 93% of respondents agreed that the recommendations are useful, and 75% considered them to be up to date. Each recommendation is scheduled for reassessment every five years to ensure alignment with emerging evidence, new clinical insights, and current standards of care for the respective rare disease. However, updates are only initiated if new, anesthesia-relevant findings become available, as frequent revisions without substantial new data may not be meaningful – particularly in the context of rare diseases, where evidence often accumulates slowly. This five-year interval is consistent with standards for clinical guideline development and appears appropriate given the typically slow pace of new research in rare disease anesthesia [21]. A minority of users considered the recommendations too lengthy, likely reflecting the time pressure common in perioperative care. Time constraints not only reduce the likelihood of full engagement, but may also negatively impact treatment quality, clinical decision-making and patient safety [22–25]. To address this, flexible formats such as concise summaries or app-based quick-access tools could offer a more practical solution for anesthetists, particularly in hospital settings where rising workload and time constraints may hinder both access to and implementation of best-practice recommendations [26].

At least, 22% of respondents reported being familiar with the OrphanAnesthesia emergency cards, despite their systematic integration into the platform only beginning in 2022. As not all recommendations currently include such cards, the low level of awareness is comprehensible. Nevertheless, among those familiar with the cards, 83% rated them as ‘very good’ or ‘good’, indicating strong perceived clinical value. As more recommendations are revised or newly developed, the proportion of conditions covered by an emergency card is expected to increase.

**Fig. 6:**
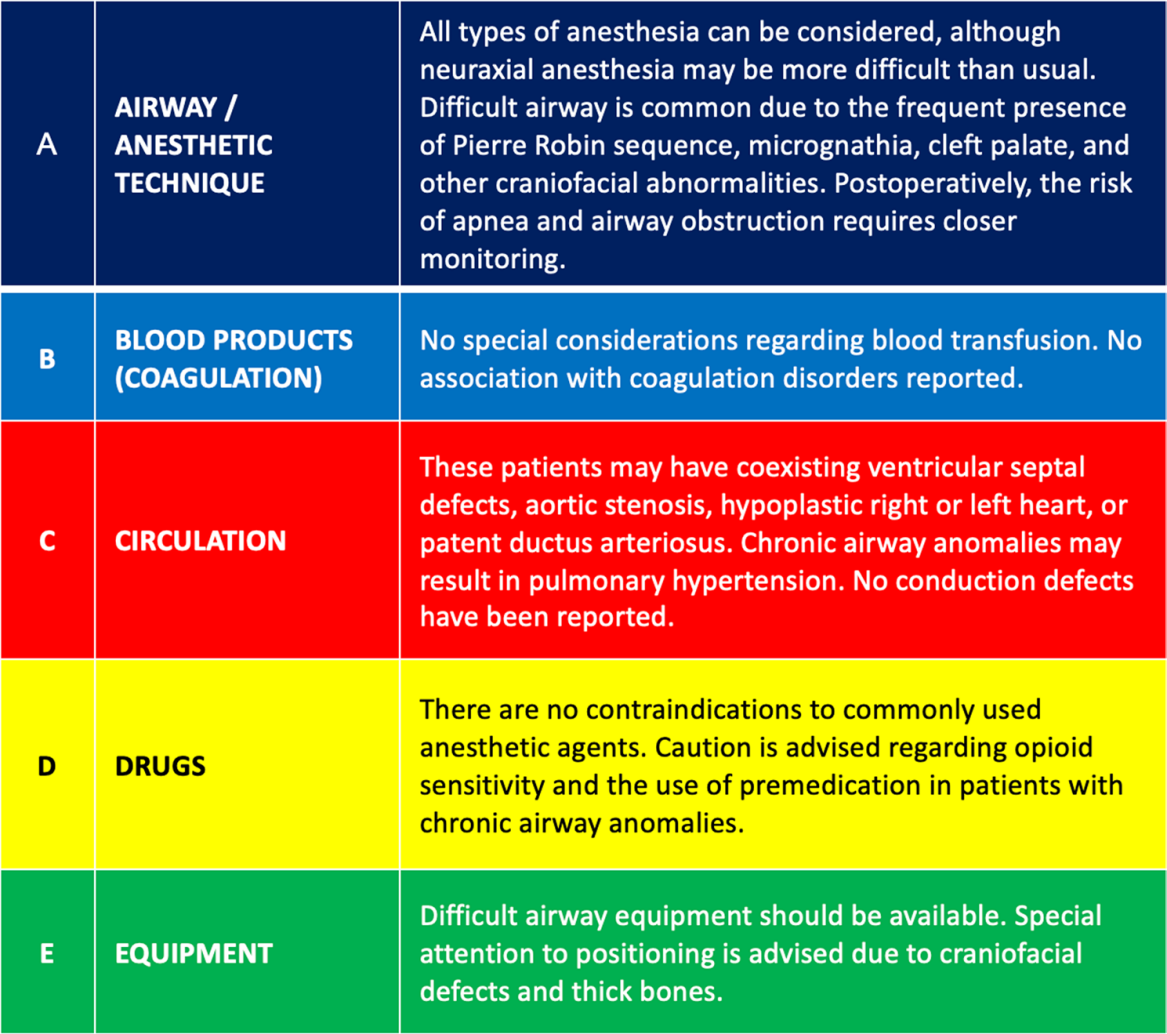
Emergency card format implemented in OrphanAnesthesia recommendations since 2022, exemplified by the Osteopathia Striata (with cranial sclerosis) syndrome, language adapted (British to American English) from [31]

Emergency cards are widely acknowledged in the literature as valuable tools for rare disease management. They provide rapid, disease-specific information and serve as communication aids between specialists and non-specialist providers [27]. However, existing emergency cards – often developed by patient advocacy groups or clinical networks – tend to offer general guidance and rarely address anesthesia-specific concerns [28–30]. The OrphanAnesthesia emergency card (Fig. 6) fills this gap by offering structured perioperative information relevant to anesthetic care [10]. Increasing awareness and systematic implementation of this tool could enhance perioperative safety in patients with rare diseases.

While general anesthesia problem cards are already available and emergency cards are useful in acute settings, a dedicated anesthesia passport could offer a more comprehensive and individualized solution in perioperative rare disease care [32]. Including data such as medications, specific dosage recommendations for the individual patient, contraindications, anesthetic risks, prior complications, as well as specific monitoring requirements and nursing considerations, such a passport could support continuity of care – particularly for patients treated across multiple institutions and by multidisciplinary teams. This type of structured documentation would be especially valuable across institutional boundaries and different hospitals, where access to prior anesthesia records may be limited or unavailable. In such settings, essential information on previous anesthetic procedures, complications, or disease-specific precautions is often missing, increasing the risk of perioperative complications. Notably, many respondents expressed – via free-text responses – a desire for OrphanAnesthesia to provide practical, protocol-like guidance akin to a ‘recipe’, outlining concrete recommendations for anesthesia management. However, such generalized guidelines are undoubtedly valuable, they may fall short in addressing the highly individualized needs and risks of patients, even among those with the same rare disease. For instance, fixed dosing regimens or standardized airway management strategies may not adequately capture the clinical heterogeneity inherent to rare disease populations. In this context, a personalized anesthesia passport – explicitly supported by many respondents – could represent a pragmatic compromise, bridging the gap between broadly applicable expert guidance and the specific, patient-level considerations essential to longitudinal patient safety and continuity of anesthetic care. The strong support expressed by respondents provides a clear mandate for the development and standardization of this tool, which could meaningfully improve anesthetic safety and decision-making in rare disease contexts.

### Limitations

This study has several limitations that must be acknowledged.

First, although technical measures such as cookies were implemented to prevent multiple submissions, the possibility of duplicate entries cannot be entirely ruled out.

Second, despite the relatively high number of respondents, selection bias is likely, as participation was voluntary. It is conceivable that anesthetists with a particular interest in rare diseases or prior familiarity with OrphanAnesthesia were more inclined to complete the survey. This may limit the representativeness of the findings.

Third, the infrequent clinical encounter with many rare diseases may lead some anesthetists to view the topic as less relevant to their daily practice. This may, in part, explain the modest overall response rate of 8.1%, which constitutes a key limitation of the study. Moreover, the demographic profile of respondents suggests a sample skewed towards more experienced practitioners working in larger hospitals or academic institutions. As such, the findings may not fully capture the perspectives of anesthetists in smaller or non-academic settings. Nevertheless, among those who accessed the survey link, a high completion rate was observed, reflecting strong engagement and yielding a substantial number of fully completed questionnaires for analysis.

Fourth, the survey was conducted exclusively among members of the DGAI and BDA. Consequently, the views of anesthetists outside these societies – including those practicing in other countries – are not represented. Given the international scope of the OrphanAnesthesia project, this constitutes an important limitation regarding the global generalizability of the results.

## Conclusion

In summary, these findings underscore the clinical relevance and increasing integration of OrphanAnesthesia among anesthetists in Germany. To further strengthen its utility, ongoing efforts should include the continued development of new recommendations and improvement of their availability. In addition, improved visibility and utilization of the anesthesia-specific emergency cards, the development of a personalized anesthesia passport, and the implementation of mobile, user-centered access tools may help expand the platform’s use among anesthetists. By continuing these efforts, OrphanAnesthesia can further contribute to enhancing patient safety and advancing more informed, individualized, and patient-centered anesthetic care in the context of rare diseases.

## Supplementary Information

Supplementary Material 1.

## Data Availability

Data are available upon reasonable request. All data relevant to the study are included in the article or uploaded as online supplemental information.

## Abbreviations

BDA: German Association of Anesthetists
DGAI: German Society for Anesthesiology and Intensive Care Medicine
EMS: Emergency medical service
EU: European Union
FDA: (United States) Food and Drug Administration
GARD: Genetic and Rare Disease Information Center
INSERM: Institut National de la Santé et de la Recherche Médicale
OMIM: Online Mendelian Inheritance in Man
STROBE: Strengthening the Reporting of Observational Studies in Epidemiology guidelines
