# Supplementary material for: Evaluation and perspectives of the OrphanAnesthesia project – a survey among anesthesiologists in Germany
Source: BMC Anesthesiol. 2026 Feb 11;26:145. doi: 10.1186/s12871-026-03665-7 (PMC12937518; doi:10.1186/s12871-026-03665-7)
Supplement: Supplementary file 1 — Supplementary Material 1. [file 12871_2026_3665_MOESM1_ESM.pdf]

Supplementary material:
Evaluation and Perspectives of the OrphanAnesthesia Project – A Survey Among Anesthesiologists in Germany

1. For how many years have you been working in anesthesia?
- Less than 5 years
- 5 - 10 years
- 11 - 20 years
- For more than 20 years

2. Please indicate your current position in your department:
- Resident
- Consultant
- Senior Consultant
- Chief position
- Other

3. In what size hospital do you work?
- Hospital with less than 299 beds
- Hospital with 300 to 499 beds
- Hospital with 500 to 799 beds
- Hospital with more than 800 beds and / or university hospital
- Anesthetist in private practice
- Freelance anesthetist
- Other workplace

4. Which patients have you mainly cared for in the past three years?
- Predominantly adult patients
- Adults and pediatric patients equally
- Predominantly pediatric patients
- No pediatric patients (in the past three years)

5. How do you currently obtain information on the anesthetic management of patients with rare diseases?
(Please select up to two preferred sources)
- Consulting colleagues or experts
- Textbooks
- Internet search engine (e.g., Google, Bing)
- OrphanAnesthesia
- Scientific databases (e.g., PubMed, Orphanet)
- Other

6. Are you familiar with the OrphanAnesthesia project website?
- Yes
- No

6a. How often have you accessed information from the OrphanAnesthesia website in the past three years?

- More than 10 times per year
- 5-10 times per year
- 1-4 times per year
- Never

6a1. Please rate individual aspects of the recommendations using the following scale:
(1 = Strongly agree, 2 = Agree, 3 = I don't know, 4 = Hardly agree, 5 = I don't agree)

	1	2	3	4	5
The recommendations are useful.					
The recommendations are clear enough.					
The recommendations comprehensively cover all aspects relevant to anesthesia.					
The recommendations are often too long.					
The recommendations are suitably up to date.					
The didactic design (e.g., graphics, flowcharts) is inadequate.					
The recommendations are scientifically well-founded.					

6a2. How helpful were the disease-specific emergency cards included in the recommendations? Please rate them using the following grading scale:

- 1 (very good)
- 2 (good)
- 3 (satisfactory)
- 4 (sufficient)
- 5 (poor)
- 6 (unsatisfactory)

7. At your current workplace, are you able to access the OrphanAnesthesia website if needed?

- Yes, via hospital computer or tablet
- No, only via my own mobile device
- No, not at all
- I don't know

8. How helpful would an OrphanAnesthesia passport be for anesthesiologists, documenting for example drugs used, possible anesthesiological particularities, problems, and solution strategies? (Available for download on the OrphanAnesthesia website)

- Very helpful
- Helpful
- Not very helpful
- Not helpful at all
- I don't know

9. Which contents of an e-mail newsletter (e.g., twice per year) about the OrphanAnesthesia project would be helpful for you?
(Please select up to two options)
- Announcement of new recommendations
- News about the project
- Case / experience reports
- Interviews with affected patients
- Interviews with disease experts
- I don't find a newsletter helpful
- Other ___________ (please specify)

10. Would an OrphanAnesthesia app influence your usage of the platform?
- Yes, I would use OrphanAnesthesia more frequently
- No, an app would not change my behavior

11. Which functions would be particularly important to you in an app?
(Please select up to 3 options)
- No fee for access
- Offline use possible
- Option to save favorites or notes
- Links to other resources (e.g., PubMed, Orphanet)
- "Emergency card" with key information on rare disease
- Other ___________ (please specify)

12. Do you believe that an OrphanAnesthesia app could increase patient safety in the operating room?
- Yes
- No
- I don't know

13. Do you have any ideas, suggestions, or recommendations for the future development of the OrphanAnesthesia project?
- Yes ___________ (please specify)
- No
- I don't know
